# Effects of trimester-specific and total gestational weight gain on children’s anthropometrics

**DOI:** 10.1186/1471-2393-14-351

**Published:** 2014-10-08

**Authors:** Otmar Bayer, Regina Ensenauer, Ina Nehring, Rüdiger von Kries

**Affiliations:** Institute for Social Paediatrics and Adolescent Medicine, Ludwig-Maximilians-Universität München, Haydnstr. 5/4. OG, 80336 Munich, Germany; Research Center, Dr. von Hauner Children’s Hospital, Ludwig-Maximilians-Universität München, Munich, Germany; Experimental Pediatrics, Department of General Pediatrics, Neonatology and Pediatric Cardiology, University Children’s Hospital, Heinrich-Heine-Universität Düsseldorf, Düsseldorf, Germany

**Keywords:** Gestational weight gain, Pregnancy, Priming, Children, Overweight, Obesity, Structural equation models

## Abstract

**Background:**

Gestational weight gain (GWG) has been shown to be a risk factor for overweight in offspring.

Aim of this study was to quantify the contributions of trimester-specific and total GWG on offspring’s BMI and waist circumference (WC). This is of interest for the design of interventions targeted at women showing a high GWG in early pregnancy.

**Methods:**

In a retrospective cohort study data on GWG (total and by trimester, exposure), a number of potential confounders, and children’s BMI z-scores and WC (outcomes) were analyzed using structural equation models to disentangle the trimester-specific direct effects of GWG and indirect effects mediated via total GWG.

**Results:**

7313 mother child pairs with a children’s mean age of 5.81 years were analyzed. Total effects (indirect + direct) of GWG (kg/week) on children’s BMI z-score and WC (cm) were observed in all trimesters, most prominently in the second. The longitudinal effect of GWG is a composite of trimester-specific direct effects (on BMI: 0.105, 0.255, 0.002, on WC: 0.538, 1.64, 0.308) and total GWG (on BMI 0.608, on WC: 1.03) at the end of pregnancy.

**Conclusions:**

Both trimester-specific priming and total GWG explained offspring’s anthropometrics. The results indicate, that reversal from additional weight gain attained early in pregnancy resulting in normal total GWG at the end of pregnancy might still contribute to a substantial reduction of offspring’s BMI and WC.

## Background

Weight gain in pregnancy is associated with different perinatal [1] and longterm outcomes in mothers and offspring [2–4], which is underlined by recommendations for gestational weight gain (GWG) by the Institute of Medicine [5]. Several studies observed associations between GWG and children’s anthropometric outcomes [6–10]. More recently, studies revisited this association subdividing the pregnancy period, raising the question, whether there are critical periods for GWG with respect to priming children’s BMI [11]. As there is a strong need for early prevention concepts in counteracting childhood overweight and obesity, it is of major interest to define the point in time when an intervention against excessive GWG should be delivered. Recommending strategies to reduce GWG for all women irrespective of their individual risk (scattergun approach) has the advantage of the earliest intervention possible but is associated with the risk of increasing inadequate GWG [12]. Targeting interventions would require to first observe the pregnant women’s weight gain in order to decide, whether they excessively gain weight or not. As a consequence, interventions would be delayed, leading to a dilemma, if later GWG would not affect the desired outcome, in this case offspring’s BMI.

We therefore revisited the research question, if there are critical periods during pregnancy with respect to GWG and programming of a risk of overweight and abdominal adiposity in the offspring. Further we aimed to elucidate to what extent these are direct effects of GWG in the respective periods, that cannot be explained by their contribution to total weight gain.

## Methods

### Participants and data sources

From October 2009 to June 2011, all children attending the mandatory school entry health examinations in 6 regions in Bavaria, both urban and rural, were invited to participate in this retrospective cohort study (PEPO: PErinatal Prevention of Obesity). Detailed information about sample recruitment and data collection has been published previously [13].

Prior to the school entry health examinations, information leaflets were sent to all families. Mothers and their children were invited to take part in the study, and a questionnaire on sociodemographic and other risk factors for childhood overweight was completed at the time of examination. Anthropometric data were collected by trained study nurses on the day of the school entry health examination. The child’s weight was measured, wearing underwear, with a calibrated electronic scale (Seca, Birmingham, UK) with an accuracy of 0.1 kg. Height was measured, without wearing shoes, with an accuracy of 0.1 cm using a stadiometer (Seca, Birmingham, UK). Waist cirumference was measured to the nearest 0.1 cm midway between the lowest rib and the top of the iliac crest according to the WHO recommendations [14]. Measurements were carried out at the end of a gentle expiration after an inelastic tape (Seca) was placed directly on the skin of the child who was standing balanced on both feet, without wearing shoes, and whose arms were hanging freely. Measurements were carried out three times and the mean was used for analysis.

Data on maternal pre-pregnancy weight and height and GWG in the first, second and third trimesters, diabetes and gestational diabetes were obtained from the “maternity pass.” In Germany, a “maternity pass” is issued to every pregnant woman at her first antenatal visit to the gynaecologist. The mothers keep the “maternity pass” as a personal document for the first and all subsequent pregnancies. The document contains health care information relevant to the pregnancy, and also includes data on serial weight measurements over the course of pregnancy documented by the consulted physician. On the day of the school entry health examination, trained study nurses copied weight-related data from the “maternity pass”.

The study protocol was approved by the ethics committee of the Ludwig-Maximilians Universität München, and signed informed consent was obtained from all participants.

### Explanatory variables

The exposures of interest were trimester-specific and total GWG. First trimester (t1) weight gain was calculated by subtracting pre-pregnancy weight from the weight measured between the first and 13th week. Second trimester (t2) weight gain was calculated by subtracting the weight measured in t1 from the weight measured between week 14 and 26. Third-trimester (t3) weight gain was calculated by subtracting the weight measured in t2 from the weight measured between week 27 and 40. Measurements outside these time frames were excluded.

The following potential confounding factors, which were ascertained by use of a questionnaire including questions from the KiGGS study [15] and a recent Bavarian study on breastfeeding [16] were used in the analysis [17]: Breastfeeding was dichotomized as “at least one month full-time without interruption” and “less than one month full-time”. Maternal smoking was dichotomized as “at no time during pregnancy” or “any time during pregnancy”. Parental SES was defined in three categories using an additive index based on maternal and paternal educational background and current type of maternal and paternal employment [18]. Maternal age and pre-pregnancy BMI were treated as continuous variables.

### Statistical analysis

**Figure 1:**
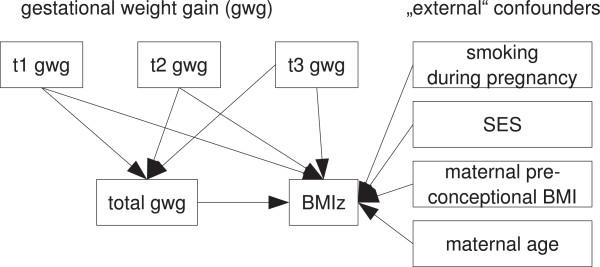
**Diagram of the structural equation model.**

To disentangle the effects of trimester-specific and total GWG (which are highly correlated due to the contribution of each trimesters’ weight gain to total GWG) on the offspring’s BMI and waist circumference in childhood, we set up a structural equation model depicted in Figure 1. Structural equation models deal with highly correlated measurements, and make it possible to obtain estimates for direct, indirect, and total effects. The indirect effect is the part of the effect mediated through other covariates on the path (e. g. GWG at t1 contributing to total GWG, which in turn affects offspring’s BMI). The total effect is the sum of direct and indirect effect. To adjust for potential confounding by parental SES, maternal smoking during pregnancy, age, pre-pregnancy BMI, these variables were included to directly affect offspring’s BMI z-score (Figure 1). Since there were no applicable reference values to convert the waist circumference data to z-scores, the untransformed values in cm were used, additionally adjusting for children’s age and sex in the model. In the BMI model the use of age and sex specific BMI z-scores inherently corrects for these two variables.

Standard errors of indirect effects were estimated using Sobel’s (1982) method [19].

## Results

**Figure 2:**
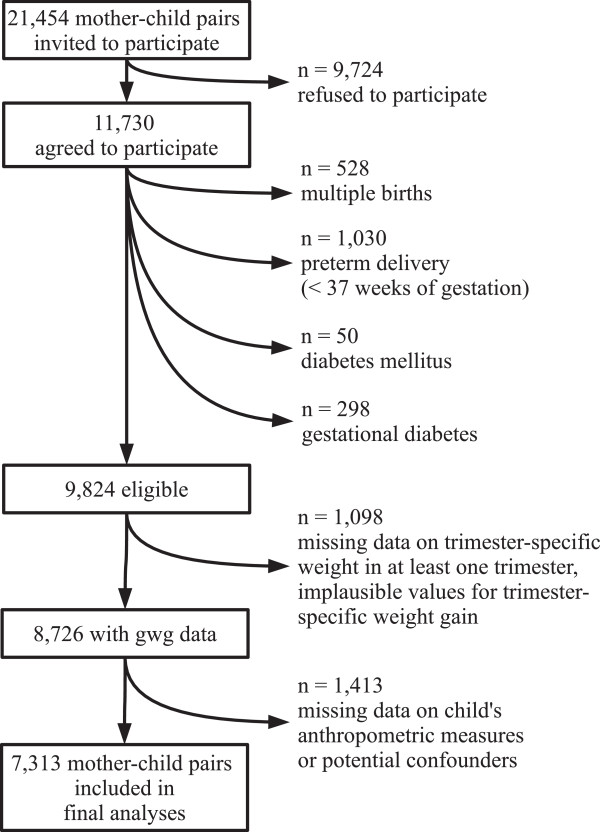
**Participants flow diagram.**

**Figure 3:**
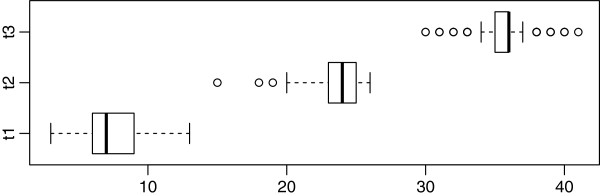
**Time (in weeks) of weight measurement during pregnancy.** Shown are the median (t1: 7, t2: 24, t3: 36) surrounded by the interquartile range (box), the most extreme values within 1.5 interquartile ranges from the box (whiskers) and outliers (circles).

The numbers of recruited and included subjects are illustrated in Figure 2. The median times of GWG measurements included were 7, 24, and 36 weeks after conception for t1, t2, and t3 (Figure 3).

Data on BMI z-score, WC and maternal GWG were available for 7769 children, additional information on external confounders (shown in Figure 1) for 7313, which served as the dataset for analysis. At follow-up the children were 5.81 ± 0.37 (mean ± std) years of age, 3580 (49.0%) were girls. Further sample description can be found in Table 1. Children excluded had a 0.06 higher BMI z-score, and a 0.38 cm higher waist circumference, which are small but statistically significant mean differences. Among the excluded participants the proportion with high parental SES and mothers who smoked during pregnancy was higher (see Table 1). The results from structural equation modeling are presented in Table 2 and can be interpreted as BMI z-score (standard deviations) per GWG (kg/week). For example, one kg more maternal weight gain during t1 results on average in a 0.2 higher BMI z-score in the child. This total effect is equally contributed by the direct and indirect effect. In summary, there is an effect of GWG on children’s BMI z-score in all trimesters, though most prominent in t2. While the effect is half a direct, and half an indirect effect in t1 and t2, for t3 it is a completely indirect effect, mediated by total GWG.

**Table 1:** **Sample description**

Variable	Mean ± std or n (percentage) eligible n = 9824	Mean ± std or n (percentage) included in model n = 7313	Mean ± std or n (percentage) excluded from model n = 2511
BMI z-score at 6 y	0.02 ± 1.04	0 ± 1.03	0.07 ± 1.1
Waist circumference in cm at 6 y	52.6 ± 4.5	52.5 ± 4.3	52.8 ± 4.9
Weekly GWG at t1 in kg	0.1 ± 0.3	0.2 ± 0.3	0.1 ± 0.4
Weekly GWG at t2 in kg	0.4 ± 0.2	0.4 ± 0.2	0.4 ± 0.2
Weekly GWG at t3 in kg	0.5 ± 0.2	0.5 ± 0.2	0.5 ± 0.2
Maternal age in years	28.9 ± 5.3	29 ± 5.1	28.7 ± 5.7
Maternal pre-pregnancy BMI in kg/m^2^	23.4 ± 4.2	23.4 ± 4.2	23.4 ± 4.4
Underweight mothers (BMI < 18.5 kg/m^2^)	465 (5.1)	343 (4.7)	122 (6.5)
Overweight mothers (BMI > 25 kg/m^2^)	2377 (25.8)	1869 (25.6)	508 (27)
Obese mothers (BMI > 25 kg/m^2^)	716 (7.8)	555 (7.6)	161 (8.6)
Breastfeeding, at least one month fulltime	6703 (73.2)	5043 (73.2)	1660 (73)
Smoking, any time during pregnancy	1162 (12)	806 (11)	356 (15.2)
Parental SES “high”/“medium”/“low”	3129 (33.2)/3688 (39.1)/2614 (27.7)	2313 (31.6)/2933 (40.1)/2067 (28.3)	816 (38.5)/755 (35.6)/547 (25.8)

**Table 2:** **Effects obtained from structural equation modeling**

Effects on BMI z-scores (i. e. std) per GWG (kg/week)			
Period	Direct effect ± stderr	Indirect effect ± stderr	Total effect ± stderr (estimated via OLS regression)
t1	0.105 ± 0.060*	0.104 ± 0.043*	0.208 ± 0.042*
t2	0.255 ± 0.116*	0.241 ± 0.100*	0.498 ± 0.064*
t3	0.002 ± 0.098	0.199 ± 0.083*	0.198 ± 0.056*
Total gwg	0.608 ± 0.252*		
**Effects on waist circumference (cm) per GWG (kg/week)**			
**Period**	**Direct effect ± stderr**	**Indirect effect ± stderr**	**Total effect ± stderr (estimated via OLS regression)**
t1	0.538 ± 0.254*	0.176 ± 0.181	0.714 ± 0.179*
t2	1.644 ± 0.489*	0.408 ± 0.421	2.055 ± 0.268*
t3	0.308 ± 0.413	0.337 ± 0.347	0.641 ± 0.237*
Total gwg	1.03 ± 1.062		

The rows in this table represent the trimester-specific effects of GWG broken down to the direct part and the part mediated via total GWG. Significant (p < 0.05) are marked by *. The first column can be interpreted longitudinally over the whole pregnancy period: a mother gaining additional weight of 1 kg/week in t1 and t2 and compensating this gain in t3 arriving at no additional total GWG attains the direct effects of t1 and t2 (0.105 + 0.255 = 0.36) accounting for an increment in offspring’s BMI of 0.36 z-scores, but avoids the effect of total GWG. A mother continuing to gain additional weight of 1 kg/week in t3 would attain the direct effects of t1 – t3 plus the effect of total GWG (0.105 + 0.255 + 0 + 0.608 = 0.97).

For waist circumference, the same pattern for the total effects was observed. However, the contribution of the direct effects appeared larger in all three trimesters, while the indirect effects could not be detected as statistically significant (alpha = 0.05).

## Discussion

Structural equation modeling of 7313 mother child pairs suggests that weight gained at any time during pregnancy directly or indirectly (mediated via total GWG) affects offspring’s anthropometrics.

A substantial proportion of the effect of the 1^st^ and 2^nd^ trimester GWG on childhood BMI is mediated by total GWG. Therefore, identification of excessive GWG early in pregnancy might not justify complacency: achieving adequate total GWG will still result in lower offspring BMI and waist circumference.

The present analysis differs in two ways from previous studies published by our group [17, 20]. 1) Here, we used continuous instead of dichotomized explanatory (kg/week instead of excessive weight gain) and outcome (BMI z-score and waist circumference instead of overweight) variables. 2) Previous analyses compared cumulative GWG to the respective trimester-specific cutoffs, thus carrying information from previous trimesters (e. g. whether GWG exceeds the cutoff in t3 also depends on GWG in t1 and t2). The structural equation modeling allows to disentangle trimester specific (direct) effects from the effect of total GWG.

In many mothers excessive GWG early in pregnancy will result in high total GWG. Indeed, as recently demonstrated 80% of the mothers with excessive GWG early in pregnancy show excessive GWG at the end of pregnancy [13]. Therefore, early GWG allows to identify women at high risk for excessive total GWG. This is important with respect to the design of preventive programs against high GWG.

Such intervention programs might be delivered to all pregnant women as close to conception as possible. As shown in recent meta analyses [1, 21] such interventions are efficacious in reducing GWG. Such a shift in GWG of the total population, however, will also increase the proportion of pregnancies with inadequate (i. e. too low) GWG, which has been shown to be associated with unintended outcomes [1, 12]. Therefore, targeted interventions at women with high early GWG would be tempting, if still efficacious with respect to reducing overweight and abdominal adiposity in the offspring.

Our data confirm a strong effect of GWG in mid pregnancy as reported by others [11]. In contrast to the findings of Anderson et al. we found a still significant effect of t3 GWG on offspring’s BMI and waist circumference. This discrepancy might reflect different time periods considered: in Anderson’s study t2 extends to week 30 (max. week 32) with t3 consequently capturing the last 10 (8) weeks of pregnancy only. In our study the t3 period was in median between the 24^th^ and 36^th^ week of pregnancy. Another important difference is given by the analysis, as in Anderson’s structural equation model the indirect effect via total GWG was not considered. This, however, is essential to disentangle potential direct and indirect effects of GWG in pregnancy: if the entire effect on offspring’s BMI were mediated by direct effects via priming early in pregnancy, efforts to normalize total GWG later in pregnancy would be ineffective with respect to children’s BMI. We could not identify any further study addressing the association between trimester-specific GWG on offspring’s BMI or overweight.

Since BMI reflects both fat and lean mass, we additionally considered children’s waist circumference as a measure of abdominal adiposity. A high waist circumference has been shown to be related to cardiovascular risk factors such as total cholesterol and blood pressure in school children [22]. We therefore repeated our analysis using waist circumference instead of BMI as outcome variable. This revealed the same pattern of total effects, although direct effects were more prominent than in the BMI analysis. Nevertheless, the conclusion for interventions remain the same: A woman gaining excess weight in the first half of pregnancy and compensating this excess gain in the second half arriving at a normal GWG contributes markedly to a lower offspring waist circumference compared to a women continuing to gain excess weight throughout pregnancy (this can be calculated similar to the example given in Table 2).

We adjusted the models for a number of confounding variables, however, such adjustment is incomplete. There were no anthropometric measurements of the father available, which is a limitation. We sought to include breastfeeding (see Explanatory variables), however this would have led to exclusion of further 432 children. Therefore, we did not include breastfeeding in our main models but conducted a sensitivity analysis including this variable, which did not substantially change the results: for t3 the direct/indirect effects ± standard errors were 0.009 ± 0.101/0.173 ± 0.085 on BMI z-score, and 0.310 ± 0.421/0.290 ± 0.354 on waist circumference. The effect of total GWG was 0.531 ± 0.261 on BMI z-score, and 0.893 ± 1.090 on waist circumference.

A strength of our study is the ascertainment of maternal weight – the primary exposure variable – from medical records on measured weight. An exception to this is the pre-pregnancy weight, which was recalled at the first antenatal visit. The imprecision introduced by this limitation could introduce a bias towards null and might contribute to the weak effect of t1 GWG. However, the self-reported pre-pregnancy weight and the first measured weight in pregnancy correlated very well (r = 0.988).

The availability of two relevant outcome variables BMI and waist circumference is a further strength. The data to compute the outcome variables were measured in a standardized way at the school entry health examination.

Our findings underline the importance of the established risk factor total GWG. Although trimester-specific weight gains allow for more precise prediction of offspring BMI, total GWG is a simpler and still reasonable predictor, as there is a considerable time-independent effect.

## Conclusion

The effect of GWG on offspring’s BMI is not confined to the first two trimesters. Effects attributable to early GWG are partially mediated by total GWG and thus may be reversed if excessive total GWG is avoided. Therefore, interventions targeted at women with excessive GWG identified in early pregnancy appear warranted.

## Abbreviations

GWG: Gestational weight gain
BMI: Body mass index
WC: Waist circumference
WHO: World Health Organisation
KiGGS: Kinder- und Jugendgesundheitssurvey (The German Health Survey for Children and Adolescents)
SES: Socio-economic status
std: Standard deviation
stderr: Standard error.
